# Ku-Band 50 W GaN HEMT Power Amplifier Using Asymmetric Power Combining of Transistor Cells

**DOI:** 10.3390/mi9120619

**Published:** 2018-11-24

**Authors:** Seil Kim, Min-Pyo Lee, Sung-June Hong, Dong-Wook Kim

**Affiliations:** Department of Radio Science and Engineering, Chungnam National University, Daejeon 34134, Korea; ksl4896@naver.com (S.K.); dignitymp20@naver.com (M.-P.L.); hsj_1006@naver.com (S.-J.H.)

**Keywords:** Ku-band, GaN high electron mobility transistor (HEMT), power amplifier, asymmetric power combining, amplitude balance, phase balance

## Abstract

In this paper, we present a Ku-band 50 W internally-matched power amplifier that asymmetrically combines the power transistor cells of the GaN high electron mobility transistor (HEMT) (CGHV1J070D) from Wolfspeed. The amplifier is designed using a large-signal transistor cell model in the foundry process, and asymmetric power combining, which consists of a slit pattern, oblique wire bonding and an asymmetric T-junction, is applied to obtain the amplitude/phase balance of the combined signals at the transistor cell combining position. Input and output matching circuits are implemented using a thin film process on a titanate substrate and an alumina substrate with the relative dielectric constants of 40 and 9.8, respectively. The pulsed measurement of a 330 μs pulse period and 6% duty cycle shows the maximum saturated output power of 57 to 66 W, drain efficiency of 40.3 to 46.7%, and power gain of 5.3 to 6.0 dB at power saturation from 16.2 to 16.8 GHz.

## 1. Introduction

Conventional radar systems used traveling wave tubes, magnetrons, or klystrons to obtain high output power, but they had the disadvantages of a high operating voltage, large size, a short lifetime, and low reliability. Recently, a GaN high electron mobility transistor (HEMT), which features a low operating voltage, easy maintenance, a small form factor, and better reliability, has been widely used in solid-state power amplifiers (SSPAs), a key component of modern radar systems [1,2,3]. In comparison with other GaAs-based or Si-based transistors, the GaN HEMT has superior electron transport, high breakdown voltage, and high thermal conductivity; therefore, GaN HEMT power amplifiers showing larger output power and better efficiency have been actively studied and published [4,5,6,7].

In this paper, we present a Ku-band internally-matched power amplifier, which uses a GaN HEMT (CGHV1J070D) bare die from Wolfspeed (Durham, NC, USA) and a thin film process for input and output matching circuits on two different substrates. For the balance of the amplitude and phase of the signals at the power-dividing and power-combining positions, we apply the asymmetric power combining of transistor cells which uses a slit pattern, oblique wire bonding and asymmetric T-junction in the input and output matching circuits.

## 2. Power Amplifier Design

### 2.1. Device Description

**Figure 1:**
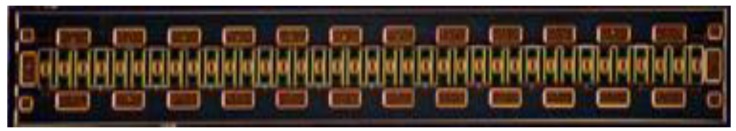
Photograph of a GaN high electron mobility transistor (HEMT) (CGHV1J070D) from Wolfspeed.

In this work, the GaN HEMT, which is fabricated on a SiC substrate with high thermal conductivity, has a 0.25-μm gate length and operates up to 18 GHz. It has a size of 800 μm × 4800 μm and a saturated output power capability of 70 W at the reference plane of its drain pad. Considering output matching loss, we chose this device for a Ku-band 50 W power amplifier. A photograph of its chip and its main performance parameters are shown in Figure 1 and Table 1 [8].

### 2.2. Device’s Optimal Source and Load Impedances

The GaN HEMT consists of 12 transistor cells and is estimated to have a total gate width of 14.4 mm [8,9]. Because a conventional large-signal model from Wolfspeed has only 4 ports, we cannot control or tune output signals from the transistor cells to achieve the balance of their amplitudes and phases using only the given model. In this work, we use the transistor cell model (r7 model) of the process design kit from the company’s foundry service and modify it to include its gate and drain pad effects obtained by 3D electromagnetic simulations.

**Figure 2:**
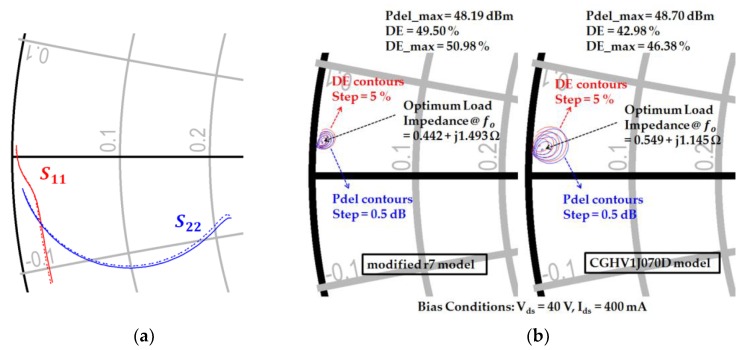
Comparison of S-parameter and load-pull simulation results of the conventional large-signal CGHV1J070D model and our modified r7 model: (**a**) S-parameter simulation; (**b**) load-pull simulation (**left**: modified r7 model, **right**: conventional CGHV1J070D model).

Figure 2 compares S-parameters and load-pull results of the conventional large-signal CGHV1J070D model and our modified r7 model. Contrary to the former, the latter has 24 ports as input and output ports and shows almost the same DC curve, maximum available gain and stability performance. As seen in Figure 2, the S-parameter and load-pull simulation results also show only a slight difference due to the parasitic gate and drain pad capacitance.

The optimum source impedance and load impedance are obtained by source-pull and load-pull simulation results of our modified r7 model using the Keysight Advanced Design System (ADS) circuit simulator (ADS 2015, Keysight, Santa Rosa, CA, USA). The simulation results show that the optimum source impedance and load impedance are Z_S_ = 0.121 − j 0.251 Ω and Z_L_ = 0.442 + j 1.493 Ω at 16.5 GHz, respectively. The obtained optimum impedances are so small that the impedance matching is very challenging in the Ku-band power amplifier design.

### 2.3. Input and Output Matching Circuit Design

**Figure 3:**
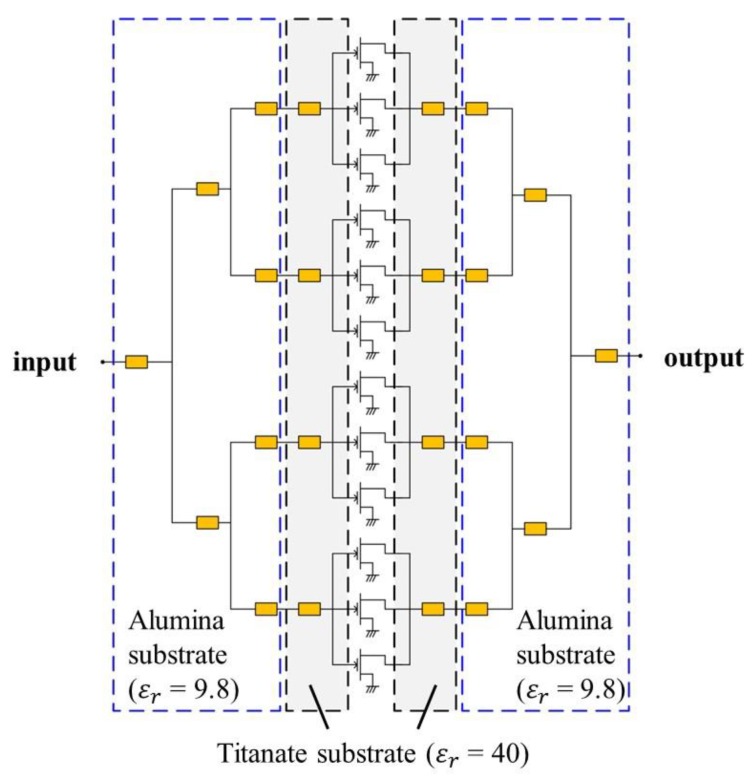
Schematic circuit diagram of the Ku-band 50 W GaN HEMT internally-matched power amplifier.

Typically, the optimum source impedance and load impedance of a high-power transistor operating at 10 GHz or above are so small that its impedance matching requires a multi-stage impedance transformer with a high impedance ratio to secure the proper operating bandwidth, which results in a large matching circuit. In the internally-matched power amplifier, which integrates transistor bare dies and thin-film matching circuits in a standard package, a large impedance-transforming matching pattern is not preferable. While maintaining the circuit performance, reducing the size of the matching circuit is essential because of the limited space available in the package. In this work, we use a titanate substrate with a high relative dielectric constant of 40 near the transistor to obtain the impedance matching trace in the low-Q region of the Smith chart and to reduce the size of the matching circuit. The overall input and output matching circuits are fabricated on two different substrates with the relative dielectric constants of 40 and 9.8, respectively. Figure 3 presents a schematic circuit diagram of our Ku-band 50 W GaN HEMT power amplifier.

The output signal of each transistor cell should be combined with the same amplitude and phase to generate high output power efficiently. Although the signal traces in the matching circuit are symmetrically placed at the combining point, the output signals experience different delays due to a combination of various bends, so the power-combining element with the symmetric matching pattern provides each transistor cell with different load impedance, not the optimum load impedance. This results in degraded power performance, rather than an optimized output power. Several design techniques are studied and published to resolve this problem [10,11,12,13].

**Figure 4:**
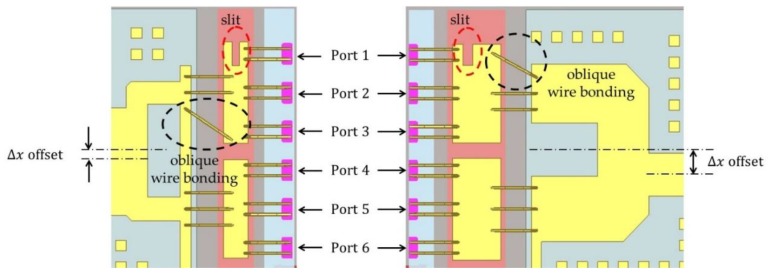
Input and output matching patterns with the slit, oblique wire bonding and asymmetric T-junction.

In this work, to obtain amplitude and phase balance at the power-combining point, we apply the asymmetric power combining, which consists of a slit pattern, oblique wire bonding and an asymmetric T-junction, to the matching circuit patterns on the gate and drain sides. The slit is fabricated on a titanate substrate and the oblique wire bonding and asymmetric T-junction are implemented on an alumina substrate. Figure 4 shows the slit, oblique wire bonding and asymmetric T-junction which are applied to 6 transistor cells. The slit is located on the side of the outer transistor cells (ports 1 to 3), and the T-junction is off-center to the inner transistor cells (ports 4 to 6). In addition to the slit and asymmetric T-junction, the oblique wire bonding improves the maximum amplitude imbalance within 0.3 dB and the maximum phase imbalance within 0.2 degree from 16 GHz to 17 GHz.

**Figure 5:**
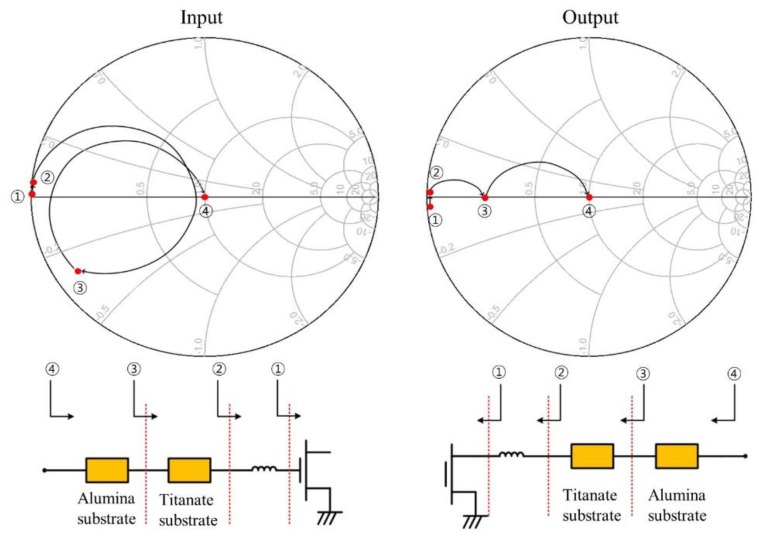
Impedance traces at the representative positions of input and output matching circuits.

**Figure 6:**
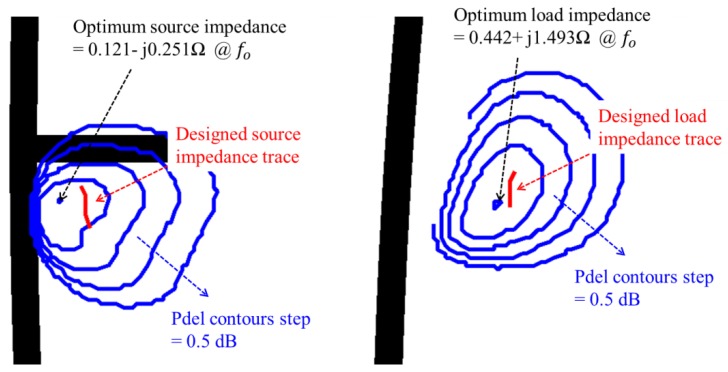
Designed source and load impedance traces overlapped on the simulated source-pull and load-pull contour plots.

Figure 5 shows impedance traces on the Smith chart which are seen at several representative positions of the input and output matching circuits. The impedance matching patterns are carefully designed to maintain their traces in the low-Q region of the Smith chart. Figure 6 compares the designed source impedance and load impedance with the optimum source impedance and load impedance. The designed impedance traces make the saturated output power be within the contour plots above 47.6 dBm.

## 3. Fabrication and Measurement

### 3.1. Power Amplifier Fabrication

**Figure 7:**
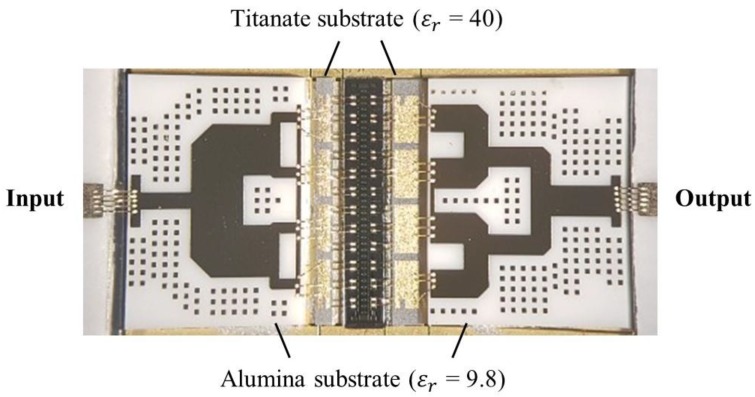
Fabricated internally-matched power amplifier using a GaN bare die and 4 input and output matching substrates.

A GaN HEMT bare die and 4 input/output matching circuits on the titanate and alumina substrates are attached to a CuW carrier using a eutectic die-attach process and silver epoxy process, to facilitate heat sinking and spreading and interconnect the power amplifier circuit to a printed circuit board (PCB) test circuit using 1-mil wedge bonding. Figure 7 shows the fabricated internally-matched power amplifier.

### 3.2. Power Amplifier Measurement

**Figure 8:**
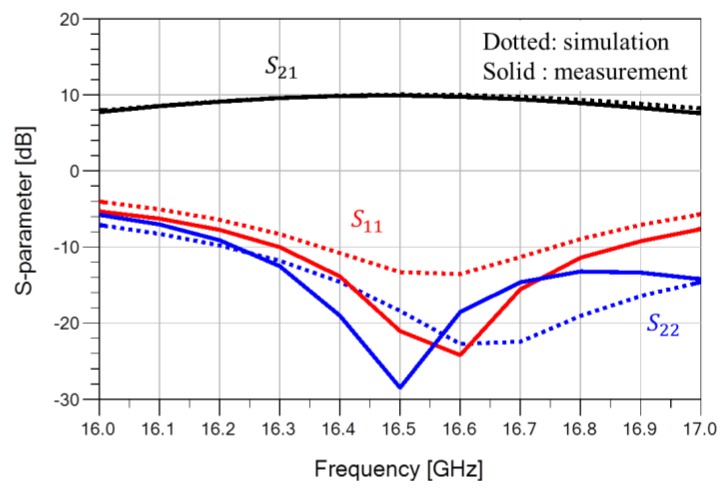
Simulated and measured S-parameter results (simulation: dotted lines, measurement: solid lines).

The performance of the fabricated GaN HEMT power amplifier was measured under the bias conditions of V_DS_ = 40 V and I_DS_ = 400 mA. Figure 8 compares the simulated (dotted lines) and measured (solid lines) S-parameter results of the power amplifier. The measured small-signal gain (S_21_) was more than 8.9 dB and the input return loss was better than 7.7 dB in the range of 16.2 to 16.8 GHz, which was in good agreement with the simulated results.

**Figure 9:**
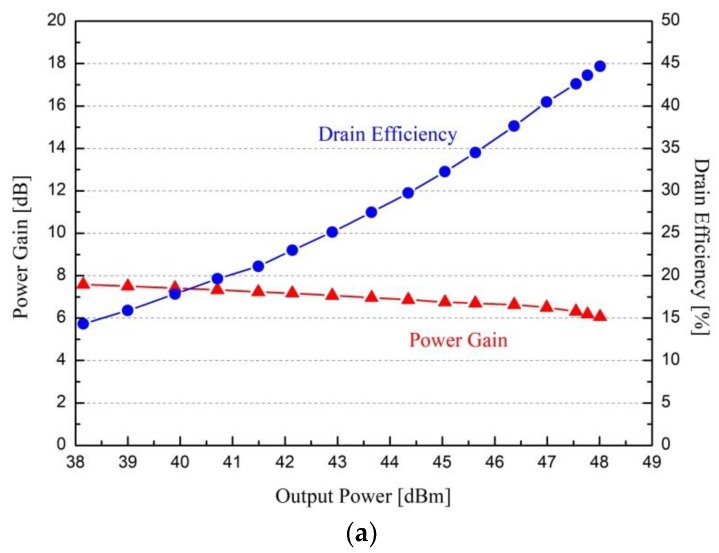

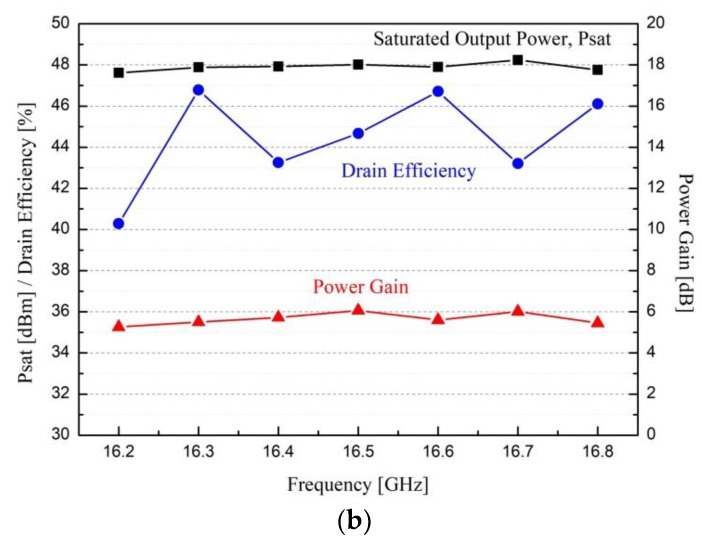
Measured output power performance results of the fabricated power amplifier at V_DS_ = 40 V and I_DS_ = 400 mA under the pulsed condition: (**a**) power gain and drain efficiency with output power at 16.5 GHz and (**b**) saturated output power, power gain and drain efficiency with input signal frequency of 16.2 to 16.8 GHz.

The output power performance of the fabricated power amplifier was measured from 16.2 to 16.8 GHz and is shown in Figure 9. Figure 9a shows the power gain and drain efficiency with the output power at 16.5 GHz and Figure 9b shows the saturated output power, power gain, and drain efficiency with the input signal frequency of 16.2 to 16.8 GHz, under the pulsed measurement with the pulse period of 330 μs and the duty cycle of 6%. The measured output power performance showed the saturated output power of 48.0 dBm (63.2 W), the power gain of 6 dB, and the drain efficiency of 44.6% at 16.5 GHz. From 16.2 to 16.8 GHz, the measured output power was 47.6 to 48.2 dBm (57 to 66 W), and the drain efficiency was 40.3 to 46.7%, while the power gain was 5.3 to 6.0 dB.

Table 2 compares the measured output power performance with previously published results of GaN power amplifiers operating in the Ku band. As seen in the table, our internally-matched power amplifier achieves comparable performance or better results in terms of output power and efficiency in comparison with the performance of other GaN power amplifiers.

## 4. Conclusions

In this work, we designed and fabricated a Ku-band GaN HEMT internally-matched power amplifier using the asymmetric power-combining of the transistor cells, which utilized a slit pattern, oblique wire bonding and an asymmetric T-junction. The asymmetric power-combining helps to obtain the amplitude and phase balance of the transistor power cells, which can increase the power of combined output signals by combining the power transistor cells in phase. The fabricated power amplifier showed the saturated output power of 57 to 66 W, the power gain of 5.3 to 6.0 dB and the drain efficiency of 40.3 to 46.7% from 16.2 GHz to 16.8 GHz under the pulsed condition. The fabricated power amplifier achieved very competitive performance for the applications of Ku-band radar systems and other high-power transmit/receive systems.

## Figures and Tables

**Table 1:** Main performance parameters of CGHV1J070D.

Parameters	Specifications
Operating frequency	10 MHz—18 GHz
Saturated output power	70 W
Power-added efficiency	60% at 10 GHz
Small-signal gain	17 dB at 10 GHz
Operating voltage	40 V
Size	800 μm × 4800 μm

**Table 2:** Comparison of our work and previously published Ku-band GaN HEMT power amplifiers.

References	Frequency (GHz)	Power Gain (dB)	P_sat_ (W)	Efficiency @ P_sat_ (%)	Drain Voltage (V)
[14]	16.0	12.8	24.2	22 ^1^	31
[15]	16.0~16.5	6.1 ^2^	8 ^2^	25 ^1,2^	8
[16]	16.2~16.8	5.0	50	30	50
[17]	14.0~14.5	5.5	50	21 ^1^	40
This work	16.2~16.8	5.3~6.0	57~66	40~47	40

^1^ Power-added efficiency; ^2^ Measured values at P_1dB_ compression point.
